# The Role of Negative-Pressure Wound Therapy in Patients with Fracture-Related Infection: A Systematic Review and Critical Appraisal

**DOI:** 10.1155/2021/7742227

**Published:** 2021-10-19

**Authors:** Susan Haidari, Frank F. A. IJpma, Willem-Jan Metsemakers, Wies Maarse, H. Charles Vogely, Alex J. Ramsden, Martin A. McNally, Geertje A. M. Govaert

**Affiliations:** ^1^Department of Trauma Surgery, University Medical Center Utrecht, Utrecht, Netherlands; ^2^Department of Trauma Surgery, University Medical Center Groningen, Groningen, Netherlands; ^3^Department of Trauma Surgery, University Hospitals Leuven, Leuven, Belgium; ^4^Department of Plastic, Reconstructive and Hand Surgery, University Medical Centre Utrecht, Utrecht, Netherlands; ^5^Department of Orthopedic Surgery, University Medical Centre Utrecht, Utrecht, Netherlands; ^6^Department of Plastic and Reconstructive Surgery, Oxford University Hospitals NHS Foundation Trust, Oxford, UK; ^7^Bone Infection Unit, Nuffield Orthopedic Centre, Oxford, UK

## Abstract

**Introduction:**

Fracture-related infection (FRI) is a severe musculoskeletal complication in orthopedic trauma surgery, causing challenges in bony and soft tissue management. Currently, negative-pressure wound therapy (NPWT) is often used as temporary coverage for traumatic and surgical wounds, also in cases of FRI. However, controversy exists about the impact of NPWT on the outcome in FRI, specifically on infection recurrence. Therefore, this systematic review qualitatively assesses the literature on the role of NPWT in the management of FRI.

**Methods:**

A literature search of the PubMed, Embase, and Web of Science database was performed. Studies that reported on infection recurrence related to FRI management combined with NPWT were eligible for inclusion. Quality assessment was done using the PRISMA statement and the Newcastle-Ottawa Quality Assessment Scale.

**Results:**

After screening and quality assessment of 775 unique identified records, eight articles could be included for qualitative synthesis. All eight studies reported on infection recurrence, which ranged from 2.8% to 34.9%. Six studies described wound healing time, varying from two to seven weeks. Four studies took repeated microbial swabs during subsequent vacuum dressing changes. One study reported newly detected pathogens in 23% of the included patients, and three studies did not find new pathogens.

**Conclusion:**

This review provides an assessment of current literature on the role of NPWT in the management of soft tissue defects in patients with FRI. Due to the lack of uniformity in included studies, conclusions should be drawn with caution. Currently, there is no clear scientific evidence to support the use of NPWT as definitive treatment in FRI. At this stage, we can only recommend early soft tissue coverage (within days) with a local or free flap. NPWT may be safe for a few days as temporarily soft tissue coverage until definitive soft tissue management could be performed. However, comparative studies between NPWT and early wound closure in FRI patients are needed.

## 1. Introduction

Fracture-related infection (FRI) [1] is a severe complication in trauma surgery. It can delay bone and soft tissue healing and lead to persistent disability, with an overall reported treatment failure (e.g., amputation of the infected limb, recurrent infection) of 4-11% [2–5]. With a reported FRI rate up to 25-30% [6] in complex injuries, this condition has both a major personal and societal impact [7, 8]. Even after surgically successful treatment of FRI, patients report lower quality of life [9]. Healthcare costs for patients with infections are significantly higher compared to noninfected patients, mostly due to prolonged hospitalization [10–12], and the fact that patients often undergo multiple surgeries. With an increasing prevalence of FRI, this indicates an upcoming challenge for healthcare systems [13].

Recently, an international expert group developed guidelines promoting a structured treatment approach for FRI, primarily based on the concept of thorough surgical debridement with dead space management, stabilization of the fracture (if needed), and robust soft tissue coverage followed by adequate antibiotic therapy [14]. Meticulous surgical excision of all nonviable tissue is however often associated with the inevitable creation of significant soft tissue defects. Since the Food and Drug Administration approval in 1995, the application of negative-pressure wound therapy (NPWT) is increasingly popular for the temporary coverage of complex traumatic wounds [15]. Using this technique, a sterile, open cell foam or gauze dressing is applied on the soft tissue defect and connected through suction tubes to a vacuum pump and liquid waste collector. A controlled negative pressure in a range from -50 to -125 mmHg can be applied constantly or intermittently [16]. In acute open traumatic wounds with fractures, coverage with NPWT is believed to prevent ongoing contamination of the wound and to help maintain a moist environment. It can therefore temporarily be applied as a bridging method until definitive wound coverage is performed [17].

However, scientific evidence on its effect on the recurrence rate and treatment of FRI is conflicting. On one hand, NPWT is claimed to remove residual bacteria and to encourage granulation tissue [18–20] and new blood vessel formation in traumatic wounds and open fractures [21], leading to faster wound healing and reduction of complications [19]. On the other hand, studies reported that NPWT does not reduce the incidence of infection compared to standard dressing techniques in open fractures [22]. Furthermore, it was demonstrated that NPWT did not reduce the bacterial load [23] of involved wounds. In fact, some authors even reported an increased bacterial colonization [24]. Also, a previous evidence-based review of NPWT states its use in “untreated osteomyelitis is contraindicated” [25]. Therefore, the aim of this systematic review is to qualitatively assess the available medical literature on the role of NPWT in the management of established FRI.

## 2. Methods

### 2.1. Literature Search Strategy

This systematic review was performed according to the guidelines recommended by the PRISMA (Preferred Reporting Items for Systematic Reviews and Meta-Analyses) statement [26] and its “Explanations and Elaboration” [27]. A computerized literature search was performed for published articles in the PubMed, Embase, and Web of Science databases. A final literature search was conducted at March 18^th^ 2021. The search string included all synonyms for “negative pressure wound therapy” and “fracture related infection,” combined by “OR/AND.” The complete syntax is shown in Appendix I. In addition, references of included studies and reviews that were found in this search strategy were screened and, if relevant, added to this analysis. Duplicates were removed, and all titles and abstracts were screened by the first author (SH) according to the following inclusion and exclusion criteria. If eligibility was uncertain after title/abstract screening, the full text was assessed to judge if the inclusion criteria were met. Remaining uncertainties were resolved in a consensus meeting with a second reviewer (GG).

### 2.2. Inclusion Criteria

Articles were included in this analysis if

**Figure 1:**
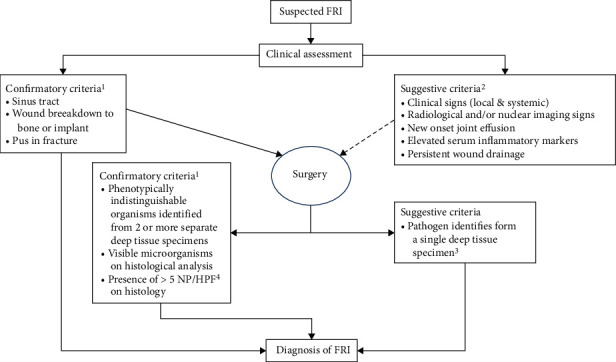
Flowchart of confirmatory and suggestive criteria visualized by McNally et al. (2020), using the definition criteria of the FRI Consensus Group [42].

- The patient population was diagnosed with FRI according to the FRI consensus definition as shown in Figure 1 [1, 28]
- NPWT was applied to the associated infected wound
- The full article was available in the English, Dutch, or German language

### 2.3. Exclusion Criteria

Articles were excluded from this analysis if

- The included study population (partly) consisted of patients with other disease entities than infected fractures (such as spondylodiscitis, diabetic feet, and pressure ulcers)
- The study population concerned animals

### 2.4. Quality Assessment

The methodological quality of the selected studies was assessed using the Newcastle-Ottawa Quality Assessment Scale (NOS) [29], a tool used for assessing the quality of nonrandomized cohort and case control studies. The NOS consists of criteria organized into three categories: selection (maximum four stars), comparability (maximum two stars), and outcome (maximum three stars). A study can be awarded a maximum of nine stars. In this systematic review, an overall NOS score cutoff of ≥7 stars was defined as high quality and <7 stars as low quality. Criteria scored “N/A” were not taken into account. Only high-quality studies were included for further analysis.

### 2.5. Data Extraction and Analysis

From the high-quality papers, the following study characteristics were extracted: bibliography (first author, year of publication, country), study design, number of inclusions, patient demographics, cause of injury, type of pathogens, NPWT duration, additional treatment, final wound closure, and follow-up duration. The primary outcome was recurrence of infection.Secondary collected outcomes were wound healing time and detection of new (morphologically different) pathogens in follow-up swabs compared to the initial swab. Due to heterogeneity of studies and lack of reported outcomes, a meta-analysis could not be performed. The data was analyzed descriptively and classified according to the quality assessment.

## 3. Results

### 3.1. Included Studies

**Figure 2:**
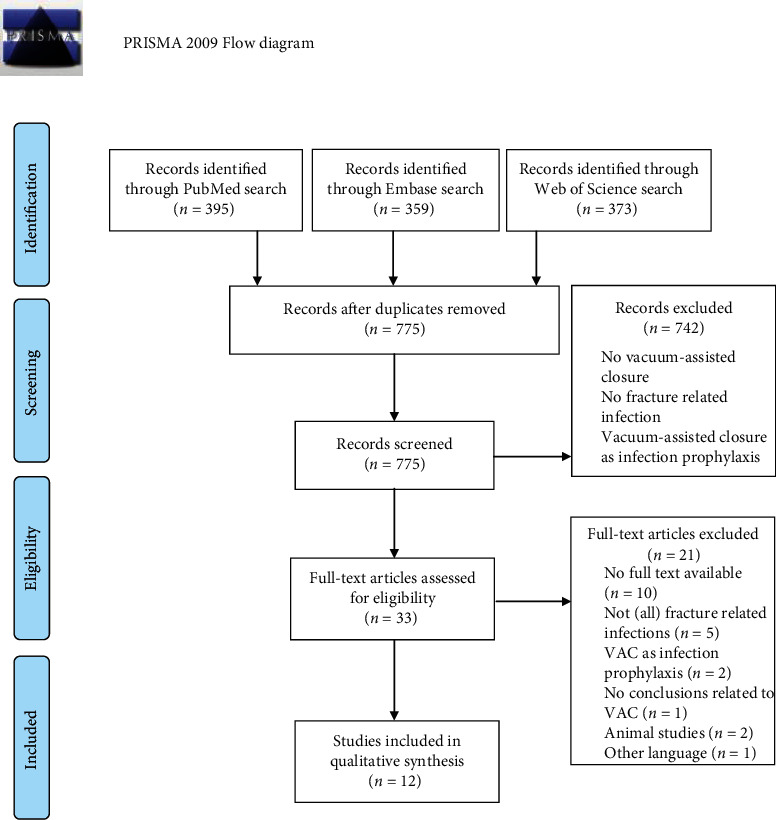


Initial database searches in PubMed (*n* = 395), Embase (*n* = 359), and Web of Science (*n* = 373) resulted in 1127 potential publications. Reference list screening of the relevant articles yielded one additional article [30]. After duplicates were removed (*n* = 352), 775 relevant articles remained for title and abstract screening which led to the exclusion of most records (*n* = 742). The 33 remaining titles were assessed for eligibility by evaluation of the full text. Finally, 12 studies were included in the qualitative synthesis. The inclusion process is based on the PRISMA guidelines [26] and summarized in the flow diagram in Figure 2.

### 3.2. Study Quality

The results of the methodological quality assessment are presented in Table 1. The quality differed between studies. Eight studies were of high quality [30–37] (≥7 stars) and four of low quality [38–41] (<7 stars). Case reports were not assessable and therefore considered as low quality. In general, the comparability criteria scored moderately due to a wide variety in outcome measures. The overall study selection and assessment of outcomes were of good quality. The scored quality criteria can be found in Appendix II.

### 3.3. Description of Study Characteristics

Study characteristics of the eight high-quality studies, published between 2009 and 2019, are shown in Table 2. Six cohort studies [31–36] and two case-control studies [30, 37] were included in this systematic review. Four of the cohort studies had a prospective [31, 34–36] and two a retrospective design [32, 33], respectively. NPWT duration varied and was often not reported. Due to small numbers of included studies, heterogeneity in applied treatment, and reported outcomes, pooling of data was not appropriate, and a meta-analysis was not possible. Hence, outcomes of the included studies [30–37] are shown in Table 3.

### 3.4. Outcome Measures

Infection recurrence was the main outcome and was reported in all papers. Average wound healing time was stated in days and reported in six papers. Four studies repeated microbial swabs at vacuum dressings changes and reported on the detection of any new pathogens during the course of treatment [30, 32, 34, 36].

#### 3.4.1. Primary Outcome

The primary outcome parameter, FRI recurrence, was measured in all studies and ranged from 2.8% to 34.9%. The recurrence rate of 34.9% was reported in the largest and qualitatively highest rated study from Izadpanah et al. (2017, *n* = 106). In this study, the definition of recurrent infection was based on the local and clinical situation at the time of operative revision and the outcome of intraoperative samples taken for microbiology [32]. Reinfected patients were distributed in two groups: one initially classed as having successful eradication of infection and one with ongoing infection since the baseline swab. Both groups received identical treatment including NPWT. The number of reinfections in the successful eradication group with NPWT was not significantly lower compared to patients who remained with an ongoing infection (*p* = 0.0764), indicating that initially deemed successful treatment with NPWT had no benefit in regards to recurrence rates compared to unsuccessful eradicated infections. Yikemu et al. (2019, *n* = 78) measured infection recurrence as main outcome and reported the second highest infection recurrence in 24.4% as pin tract infection in the course of treatment of traumatic tibial osteomyelitis [35] with Ilizarov bone transport. There is no description on how pin tract infection was diagnosed, whether it was a pin tract infection only or whether it was an expression of a more extensive FRI recurrence or neither how it was resolved. Diefenbeck et al. (2011, *n* = 43) included patients with posttraumatic osteomyelitis of the extremities and noted six recurrences (19.3%) after 3.4 years on average [36]. Four out of these six patients had negative, and two patients had positive bone biopsies before secondary closure.

Tan et al. (2011, *n* = 35) reported a lower recurrence rate after treatment with NPWT. This study monitored infection by local symptoms, fever, and blood parameters. In the NPWT group, one reinfection was found after one month (2.8%), compared to seven (20.6%) in the conventional dressing group [37]. Additionally, Timmers et al. (2009, *n* = 30) found 3 cases of clinical infection recurrence (10%), which was significantly lower compared to the control group with conventional dressings, with an average time to recurrence of 8 months [30]. In the study of Kollrack et al. (2012, *n* = 7), patients who were treated with initial surgical debridement, followed by NPWT, were followed through standard radiographic scans after swabs were negative [34]. In one patient (14.3%), radiographic pseudoarthrosis was seen, due to nonunion of the medial malleolus after a trimalleolar dislocated ankle fracture. However, since most conventional radiological imaging techniques are an arbitrary method for outcome surveillance in this population, significance of this finding in regards to FRI recurrence is unclear [42].

#### 3.4.2. Secondary Outcomes

The first secondary outcome, time to wound healing, was stated in six out of eight studies but was measured in various ways. Izadpanah et al. (2017) did not describe wound healing time [32] but compared mean stay at intensive care unit (ICU) and number of lavages until assumed eradication of FRI between patients with believed cure of infection and patients with no cure of infection. No differences were found in ICU treatment, but surprisingly, more lavages were done in the assumed cured group (*p* = 0.0431). Yikemu et al. (2019) defined wound healing, with a reported average of 35 days, as complete wound coverage with granulation tissue and no visible signs of infection [35]. Again, this is an arguable outcome measure. Wound coverage with granulation tissue only is not the robust soft tissue envelop with full epithelialization that is usually pursued [43] in FRI, and ongoing infection cannot be excluded despite a granulating wound. Deng et al. (2014) removed the NPWT device after a mean of 5 weeks, with complete coverage of the bone graft surface with granulation tissue and reported this as wound healing time [33]. In this rather small retrospective series, the mean number of vacuum dressing placements was 3.6 (range: 2-8), and the average total length of hospitalization was 3.6 months (range: 2-8). Kollrack et al. (2012) and Tan et al. (2011) defined end of treatment as the point at which a dry, clean wound was achieved, respectively, after a mean of 54.4 [34] and 9.2 [37] days. Diefenbeck et al. (2011) performed 9.8 debridements on average, until eradication of infection. Despite the absence of macroscopic infection, pathogens were still found in biopsies in 15 out of 43 patients [36]. Timmers et al. (2009) reported a mean duration of NPWT of 22.4 days per executed osteomyelitis treatment [30]. Treatment was stopped when either two consecutive swabs were sterile or when enough new granulation tissue had formed to permit successful delayed primary closure or split-thickness skin grafting.

The results of repeated microbial swabs taken at later vacuum dressing changes were reported in only four out of eight studies [30, 32, 34, 36]. Initial swabs were taken before treatment during first surgical debridement, in all four studies. In Kollrack et al. (2012), initial wound swabs showed 71.4% *S. aureus* and 28.5% *Enterococcus* species [34]. All swabs were negative during the second vacuum dressing change. In the study of Diefenbeck et al. (2011), a bone specimen was taken for microscopic analysis after each revision [36]. Initial specimens showed mostly *S. aureus* (52.4%) and *Enterococcus* (21.4%). No polymicrobial infections or detection of new (morphological different) pathogens were reported. In the study of Timmers et al. (2009), deep wound swabs were taken during dressing changes, every 3-4 days [30]. The NPWT was ended if two consecutive swabs were negative or showed skin bacteria only. In total, 72 bacterial species were identified, including *S. aureus* (40.3%) and *E. cloacae* (12.5%). Thirteen patients were identified with a polymicrobial infection. Although three recurrences occurred, no new pathogens were identified. In the study of Izadpanah et al. (2017), in 87% of all patients, bacteria were isolated during primary lavage, with the largest groups consisting of *S. aureus* (39.6%) and *Staphylococcus epidermis* (11.3%). A polymicrobial infection was present in 20% of the cases. The primary presence of a pathogen did not influence treatment failure (*p* = 0.2016); although, it increased the risk of losing the implant before fracture consolidation (*p* = 0.0413). The pathogen profile changed under therapy in 23% of all cases. It is unclear whether this also concerned increased antibiotic resistance or not. Izadpanah et al. (2017) stated that the identification of new pathogens during treatment does increase the risk for treatment failure (*p* = 0.0047).

## 4. Discussion

The aim of this study was to qualitatively assess the current literature on the role of NPWT in FRI treatment with focus on recurrence rate, the detection of new pathogens at subsequent dressing changes, and time to wound healing.

Despite the claim that NPWT for the management of soft tissue defects can contribute to fast and safe infection control [15, 44], based on the best available evidence presented in this paper, the overall infection recurrence of FRI treated with NPWT ranges from 2.8% up to 34.9% [30–37]. In this perspective, several caveats should be considered. Not all studies reported on how infection recurrence was diagnosed, differences in soft tissue defects were not classified in FRI, and groups were heterogeneous in regards to clinical presentation and location of FRI. Izadpanah et al. (2017) published the highest incidence of infection recurrence (34.9%) [32]. This study consisted of the largest study population (*n* = 106) and yielded the highest quality assessment score. In general, inconsistent methods were used to diagnose FRI, such as debatable clinical infection parameters or imaging techniques [1, 42]. For instance, Diefenbeck et al. (2011) performed final wound closure after the absence of macroscopic infection [36], and Timmers et al. (2009) stopped treatment based on negative swaps [30], while Izadpanah et al. (2017) used a combination of clinical features and intraoperative samples for microbiological assessment [32], which is a more reliable diagnostic method [1, 45]. Overall, recurrence rates of FRI after treatment with NPWT are widespread and inconsistent.

Other findings were the high number of reported reoperations with the sole purpose of vacuum dressing changes (with means ranging from 1 to 10 changes) and the long period of hospitalization (with reported means ranging from 1.5 to 8 weeks). This does not support the view that NPWT encourages rapid wound healing. Also, it will increase the cost of care in this, already costly, disease.

Although scientific evidence on the use of NPWT in case of FRI is scarce, data focusing on the use of NPWT in open fractures is abundant. With earlier studies showing promising results, many subsequent trials could not replicate the initial positive outcomes. The recent WOLFF randomized trial, for example, demonstrated that there was no benefit of NPWT compared to standard wound care in 460 patients with an open fracture of the lower limb [46]. Also, a systematic review, based on seven randomized controlled trials, found that there is moderate evidence that there is no difference between NPWT and standard care in open fractures with respect to wound healing [47]. Furthermore, the FLOW trial reported an increased infection rate in all types of open fracture wounds after treatment with NPWT [48]. This evidence applies to the primary care of open fractures, which is rather different to established FRI with a soft tissue defect. The initial enthusiasm for NPWT in open fractures may not be appropriate in FRI management.

The average time needed for wound healing reported in this review ranged from 1.5 to 7 weeks [30, 33–37]. Kollrack et al. (2012) reported the highest mean of 54 (±8) days, probably since their included patients were diagnosed with angiopathy with compromised local microcirculation [34]. Nonetheless, their reported wound healing time of approximately five weeks was considered to be promising compared to the ≥12 weeks for traditional open bone grafting (the so-called Papineau technique) [49, 50]. Having said this, the Papineau technique is nowadays rarely performed due to the negative side effects related to the long period of open wound care with exposed bone grafts.

It is argued that NPWT can significantly reduce the wound surface area [39, 41, 51] and provide stable wound healing [52]. Although this might well apply for soft tissue defects only, in case of FRI, expert opinion strongly advises against the prolonged use of NPWT in this patient population [53]. The current opinion is to aim for robust and early soft tissue coverage by local or free tissue transfers to prevent further contamination and to promote tissue vascularization for antibiotic delivery and bone healing [54–56]. Experts state that the longer final coverage is delayed by NPWT, and the more wound edges and surrounding soft tissues will become negatively affected. Tissue formed under NPWT can evolve into more rigid and difficult-to-handle scar and granulation tissue which can hamper the final reconstruction for both muscle and fasciocutaneous flap inset [57]. Also, the formed granulation tissue is often of poor quality. It may need to be removed at the time of definitive reconstruction, negating any benefit from the period of NPWT [56]. Based on the results of this review, we can add the argument of high FRI recurrence rates (up to 34.9%) to this opinion.

*S. aureus* is the most common bacterium causing FRI in the studies included in this review, followed by *S. epidermidis* and *E. faecalis* [30, 32, 34, 58, 59]. This is in line with the common pathogens usually identified in FRI [60, 61]. Four included studies stated that NPWT can help infectious wounds heal regardless of the microbial characteristics [30, 34, 62, 63]. However, Izadpanah et al. (2017) stated that if new pathogens were identified during NPWT, the risk of treatment failure rose significantly [32]. These results concur with findings from a previous randomized trial by Mouës et al. (2004), who found that NPWT creates a shift with a decrease in nonfermented negative rods to an increase in *S. aureus* [23] in 54 patients who underwent either open wound management or NPWT before surgical closure of their wounds. Other included studies did not report a change in microbial strains [30, 34, 36]. To summarize, differing outcomes have been reported; therefore, more research targeting this topic is needed to understand the exact pathogen evolution in FRI patients undergoing NPWT.

In future research on the optimal management of soft tissue defects in FRI patients, it is essential that prospective clinical studies with large sample sizes and validated patient-reported outcome measures become available. Furthermore, it is necessary to include a grading system considering differences in soft tissue defects, such as the BACH classification [64]. Besides, diagnostics of FRI should not rely on swab cultures, since better techniques are well established [45]. A special focus on infection recurrence and utilization of medical care is needed, to reliably compare the results of NPWT with other wound closure techniques such as local or free tissue transfers in this patient category.

## 5. Limitations

Firstly, the main disadvantage of this systematic review is the limited number of high-quality studies on FRI treatment with NPWT. Secondly, there was a lack of uniformity in NPWT use and additional treatment, final wound closure, and measured outcomes between the included studies. Also, included studies sampled with swabs and the total number of patients were low while the variation between studies was wide. Therefore, this review could only provide limited evidence.

## 6. Conclusion

This review provides an assessment of current literature on the role of NPWT in the management of soft tissue defects in patients with FRI. Due to the lack of uniformity in included studies, conclusions should be drawn with caution. Currently, there is no clear scientific evidence to support the use of NPWT as definitive treatment in FRI. At this stage, we can only recommend early soft tissue coverage (within days) with a local or free flap. NPWT may be safe for a few days as temporarily soft tissue coverage until definitive soft tissue management could be performed. However, comparative studies between NPWT and early wound closure in FRI patients are needed.

## Figures and Tables

**Table 1:** 

Criteria		*Patro* (*N* = 1)	Li (*N* = 18)	Yikemu (N =78)	*Zhang* (*N* = 1)	Izadpanah (*N* = 106)	Raj (*N* = 20)	*Chang* (*N* = 1)	Deng 2014 (*N* = 15)	Kollrack (*N* = 7)	Tan (*N* = 35)	Diefenbeck (*N* = 43)	Timmers (*N* = 30)
Selection	1		∗	∗		∗	∗		∗	∗	∗	∗	∗
	2		N/A	∗		∗	N/A		N/A	N/A	∗	N/A	∗
	3		∗	∗		∗	∗		∗	∗	∗	∗	∗
	4		∗	∗		∗	∗		∗	∗	∗	∗	∗
Comparability	1a		∗	×		∗	∗		∗	∗	∗	∗	∗
	1b		×	×		∗	×		∗	∗	∗	∗	∗
Exposure	1		∗	∗		∗	×		∗	∗	∗	∗	∗
	2		∗	∗		∗	∗		∗	N/A	∗	∗	∗
	3		∗	∗		∗	∗		∗	∗	∗	∗	∗
Total		N/A	7	7	N/A	9	6	N/A	8	7	9	8	9

Patro, Zhang and Chang: Quality assessment using the Newcastle-Ottawa Quality Assessment Scale was not possible for case reports.

**Table 2:** 

Author	Year	Country	Study design	*N*	Patient demographics	Cause of injury	Pathogens	NPWT duration (days)	Additional treatment	Final wound closure	Follow up (months)	Quality^a^
Li et al. [31]	2019	China	Prospective longitudinal	18	14-57 yr, 67% ♂, no comorbidities	61% traffic, 39% crush	Monomicrobial	Unknown	Antibiotic bone cement, systemic antibiotics	Skin flap transplant	29.7	High 7/9
Yikemu et al. [35]	2019	China	Prospective longitudinal	78	44.5 ± 10.5 (23-68) yr, 67% ♂, no comorbidities	69% traffic, 16% high fall, 10% crush, 5% other	Unknown	Unknown	Ilizarov bone transport technique + antibiotic treatment	Unknown	18.9	High 7/9
Izadpanah et al. [32]	2017	Germany	Retrospective longitudinal	106	54 yr (SD 19), 73% ♂, comorbidities	Unknown	20% polymicrobial, 67% monomicrobial (5.6% resistant), 13% unknown	Unknown	Systemic antibiotics	78% secondary suture/mesh graft, 22% muscle flap	>12	High 9/9
Deng et al. [33]	2014	China	Retrospective longitudinal	15	44.5 (24-68) yr, 60% ♂, comorbidities unknown	Motor vehicle accidents	93.3% monomicrobial (6.7% resistant), 6.7% polymicrobial	25.2 (14-56)	Systemic antibiotics	93.3% granulation tissue, 6.7% skin graft	22.6	High 8/9
Kollrack et al. [34]	2012	Germany	Prospective longitudinal	7	63.14 ± 4.41 yr, 29% ♂, comorbidities	Unknown	Monomicrobial	54.43 ± 7.74	Unknown	Mesh grafting	N/A	High 7/9
Tan et al. [37]	2011	China	Retrospective longitudinal case-control	35 (33)	43.4 (18-82) yr, 74% ♂, comorbidities unknown	Unknown	Monomicrobial (9.4% resistant)	9.2 (4-12)	Antibiotic treatment	47.2% muscle flap, 52.8% secondary closure	15	High 9/9
Diefenbeck et al. [36]	2011	Germany	Prospective longitudinal	43	50.7 (19-96), comorbidities unknown	Unknown	Monomicrobial (14.3% resistant)	13.5 (10-16)	Systemic antibiotics	72.1% secondary closure, 14.0% skin graft, 14.0% muscle flap	32-51	High 8/9
Timmers et al. [30]	2009	NL	Retrospective longitudinal case-control	30 (94)	52 (26-81) yr, 47% ♂, 60% comorbidities	Traumatic	43.3% polymicrobial, 56.7% monomicrobial	22.4 (6-60)	Local antiseptic fluid	Unknown	43-89	High 9/9

^a^Quality assessment was performed using the Newcastle-Ottawa Quality Assessment Scale (see Table 1).

**Table 3:** 

Author	Year	*N*	Infection recurrence	Wound healing time (days)	Pathogen changes	Quality^a^
Li et al. [31]	2019	18	5.6%	Unknown	Unknown	High 7/9
Yikemu et al. [35]	2019	78	24.4%	35	Unknown	High 7/9
Izadpanah et al. [32]	2017	106	34.9%	Unknown	23%	High 9/9
Deng et al. [33]	2014	15	13.3%	35 (21-70)	Unknown	High 8/9
Kollrack et al. [34]	2012	7	14.3%	54.4 ± 7.7	0%	High 7/9
Tan et al. [37]	2011	35	2.8%	9.2 (4-12)	Unknown	High 9/9
Diefenbeck et al. [36]	2011	43	19.3%	13.5 (10-16)	0%	High 8/9
Timmers et al. [30]	2009	30	10%	22.4 (6-60)	0%	High 9/9

^a^Quality assessment was performed using the Newcastle-Ottawa Quality Assessment Scale (see Table 1).

## Supplementary Materials

Supplementary 1 Appendix I: syntax.

Supplementary 2 Appendix II: quality assessment.

## References

[1] Govaert G. A. M., Kuehl R., Atkins B. L. (2020). Diagnosing fracture-related infection: current concepts and recommendations. *Journal of Orthopaedic Trauma*.

[2] Bose D., Kugan R., Stubbs D., McNally M. (2015). Management of infected nonunion of the long bones by a multidisciplinary team. *The Bone & Joint Journal*.

[3] Kanakaris N. K., Psarakis S., Chalidis B., Kontakis G., Giannoudis P. V. (2009). Management of pelvic instability secondary to chronic pyogenic sacroiliitis: case report. *Surgical Infections*.

[4] Ochsner P. (2016). Infections of the musculoskeletal system. Basic principles, prevention, diagnosis and treatment. *Swiss Orthop. Swiss Soc. Infect. Dis. Expert Gr.*.

[5] Bezstarosti H., van Lieshout E. M. M., Voskamp L. W. (2019). Insights into treatment and outcome of fracture-related infection: a systematic literature review. *Archives of Orthopaedic and Trauma Surgery*.

[6] Papakostidis C., Kanakaris N. K., Pretel J., Faour O., Morell D. J., Giannoudis P. V. (2011). Prevalence of complications of open tibial shaft fractures stratified as per the Gustilo-Anderson classification. *Injury*.

[7] Saleeb H., Tosounidis T., Papakostidis C., Giannoudis P. V. (2019). Incidence of deep infection, union and malunion for open diaphyseal femoral shaft fractures treated with IM nailing: a systematic review. *The Surgeon*.

[8] O’Hara N. N., Mullins C. D., Slobogean G. P., Harris A. D., Kringos D. S., Klazinga N. S. (2021). Association of Postoperative Infections after Fractures with Long-term Income among Adults. *JAMA Network Open*.

[9] Walter N., Kerschbaum M., Pfeifer C. (2021). Long-term patient-related quality of life after successfully treated aseptic non-unions of the long bones. *Injury*.

[10] Olesen U. K., Pedersen N. J., Eckardt H. (2017). The cost of infection in severe open tibial fractures treated with a free flap. *International Orthopaedics*.

[11] Metsemakers W. J., Smeets B., Nijs S., Hoekstra H. (2017). Infection after fracture fixation of the tibia: analysis of healthcare utilization and related costs. *Injury*.

[12] Thakore R. V., Greenberg S. E., Shi H. (2015). Surgical site infection in orthopedic trauma: A case-control study evaluating risk factors and cost. *Journal of Clinical Orthopaedics and Trauma*.

[13] Walter N., Rupp M., Lang S., Alt V. (2021). The epidemiology of fracture-related infections in Germany. *Scientific Reports*.

[14] Metsemakers W. J., Fragomen A. T., Moriarty T. F. (2020). Evidence-based recommendations for local antimicrobial strategies and dead space management in fracture-related infection. *Journal of Orthopaedic Trauma*.

[15] Morykwas M. J., Argenta L. C., Shelton-Brown E. I., McGuirt W. (1997). Vacuum-assisted closure: a new method for wound control and Treatment. *Annals of Plastic Surgery*.

[16] Morykwas M. J., Faler B. J., Pearce D. J., Argenta L. C. (2001). Effects of varying levels of subatmospheric pressure on the rate of granulation tissue formation in experimental wounds in swine. *Annals of Plastic Surgery*.

[17] Kim J.-H., Lee D.-H. (2019). Negative pressure wound therapy vs. conventional management in open tibia fractures: systematic review and meta-analysis. *Injury*.

[18] Pollak A. N. (2010). Use of negative pressure wound therapy during aeromedical evacuation of patients with combat-related blast injuries. *Journal of Surgical Orthopaedic Advances*.

[19] Hunter J. E., Teot L., Horch R., Banwell P. E. (2007). Evidence-based medicine: vacuum-assisted closure in wound care management. *International Wound Journal*.

[20] Blum M. L., Esser M., Richardson M., Paul E., Rosenfeldt F. L. (2012). Negative pressure wound therapy reduces deep infection rate in open tibial fractures. *Journal of Orthopaedic Trauma*.

[21] Labler L. (2009). Vacuum-assisted closure therapy increases local interleukin-8 and vascular endothelial growth factor levels in traumatic wounds. *The Journal of Trauma*.

[22] Costa M. L., Achten J., Bruce J. (2018). Negative-pressure wound therapy versus standard dressings for adults with an open lower limb fracture: the WOLLF RCT. *Health Technology Assessment*.

[23] Mouës C. M., Vos M. C., Van Den Bemd G. J. C. M., Stijnen T., Hovius S. E. R. (2004). Bacterial load in relation to vacuum-assisted closure wound therapy: a prospective randomized trial. *Wound Repair and Regeneration*.

[24] Weed T., Ratliff C., Drake D. B. (2004). Quantifying bacterial bioburden during negative pressure wound Therapy. *Annals of Plastic Surgery*.

[25] Birke-Sorensen H., Malmsjo M., Rome P. (2011). Evidence-based recommendations for negative pressure wound therapy: Treatment variables (pressure levels, wound filler and contact layer) - Steps towards an international consensus. *Journal of Plastic, Reconstructive & Aesthetic Surgery*.

[26] Moher D., Liberati A., Tetzlaff J., Altman D. G., for the PRISMA Group (2009). Preferred reporting items for systematic reviews and meta-analyses: the PRISMA statement. *BMJ*.

[27] Liberati A., Altman D. G., Tetzlaff J. (2009). The PRISMA statement for reporting systematic reviews and meta-analyses of studies that evaluate health care interventions: explanation and elaboration. *PLoS Medicine*.

[28] Metsemakers W. J., Morgenstern M., McNally M. A. (2018). Fracture-related infection: a consensus on definition from an international expert group. *Injury*.

[29] Stang A. (2010). Critical evaluation of the Newcastle-Ottawa scale for the assessment of the quality of nonrandomized studies in meta-analyses. *European Journal of Epidemiology*.

[30] Timmers M. S., Graafland N., Bernards A. T., Nelissen R. G. H. H., van Dissel J. T., Jukema G. N. (2009). Negative pressure wound treatment with polyvinyl alcohol foam and polyhexanide antiseptic solution instillation in posttraumatic osteomyelitis. *Wound Repair and Regeneration*.

[31] Li J., Zhang H., Qi B., Pan Z. (2019). Outcomes of vacuum sealing drainage treatment combined with skin flap transplantation and antibiotic bone cement on chronic tibia osteomyelitis: a case series study. *Medical Science Monitor*.

[32] Izadpanah K., Hansen S., Six-Merker J., Helwig P., Südkamp N. P., Schmal H. (2017). Factors influencing treatment success of negative pressure wound therapy in patients with postoperative infections after Osteosynthetic fracture fixation. *BMC Musculoskeletal Disorders*.

[33] Deng Z., Cai L., Jin W., Ping A., Wei R. (2014). One-stage reconstruction with open bone grafting and vacuum-assisted closure for infected tibial non-union. *Archives of Medical Science*.

[34] Kollrack Y. B. M., Moellenhoff G. (2012). Infected Internal Fixation after Ankle Fractures--A Treatment Path. *The Journal of Foot and Ankle Surgery*.

[35] Yikemu X., Tuxun A., Nuermaimaiti M., Abudukeyimu A., Shayiti A. (2019). Effects of vacuum sealing drainage combined with ilizarov bone transport technique in the treatment of tibial traumatic osteomyelitis. *Medical Science Monitor*.

[36] Diefenbeck M., Mennenga U., Gückel P., Tiemann A. H., Mückley T., Hofmann G. (2011). Vakuumtherapie bei akuter postoperativer Osteitis. *Zeitschrift für Orthopädie und Unfallchirurgie*.

[37] Tan Y., Wang X., Li H. (2011). The clinical efficacy of the vacuum-assisted closure therapy in the management of adult osteomyelitis. *Archives of Orthopaedic and Trauma Surgery*.

[38] Zhang H., Li Q. (2018). Improved vacuum sealing drainage for treatment of surgical site infection following posterior spinal internal fixation. *Medicine*.

[39] Raj M., Gill S. P. S., Sheopaltan S. K. (2016). Evaluation of vacuum assisted closure therapy for soft tissue injury in open musculoskeletal trauma. *Journal of Clinical and Diagnostic Research*.

[40] Chang C., Chan H., Lim S., Khoo E., O Z. (2014). Negative pressure wound therapy in infected wound following posterior spinal instrumentation using simple self-assembled system: a case report. *Malaysian Orthopaedic Journal*.

[41] Patro B. P., Khuntia S., Sahu N. K., Das G., Patra S. K. (2020). Negative pressure wound therapy assisted closure: an effective mode of management for infected and contaminated wound with non-union fracture femur. *Cureus*.

[42] McNally M., Govaert G., Dudareva M., Morgenstern M., Metsemakers W. J. (2020). Definition and diagnosis of fracture-related infection. *EFORT Open Reviews*.

[43] Metsemakers W. J. (2021). Fracture-related outcome study for operatively treated tibia shaft fractures (F.R.O.S.T.): registry rationale and design. *BMC Musculoskelet. Disord.*.

[44] Armstrong D. G., Lavery L. A. (2005). Negative pressure wound therapy after partial diabetic foot amputation: a multicentre, randomised controlled trial. *The Lancet*.

[45] McNally M., Sousa R., Wouthuyzen-Bakker M. (2021). The EBJIS definition of periprosthetic joint infection. *The Bone & Joint Journal*.

[46] Costa M. L., Achten J., Bruce J. (2018). Effect of negative pressure wound therapy vs standard wound management on 12-month disability among adults with severe open fracture of the lower Limb. *JAMA*.

[47] Iheozor-Ejiofor Z., Newton K., Dumville J. C., Costa M. L., Norman G., Bruce J. (2018). Negative pressure wound therapy for open traumatic wounds. *Cochrane Database of Systematic Reviews*.

[48] The FLOW Investigators (2015). A trial of wound irrigation in the initial management of open fracture wounds. *The New England Journal of Medicine*.

[49] Saleh M., Kreibich D. N., Ribbans W. J. (1996). Circular frames in the management of infected tibial non-union: a modification of the Papineau technique. *Injury*.

[50] Panda M., Ntungila N., Kalunda M., Hinsenkamp M. (1998). Treatment of chronic osteomyelitis using the Papineau technique. *International Orthopaedics*.

[51] Mouës C. M., Heule F., Hovius S. E. R. (2011). A review of topical negative pressure therapy in wound healing: sufficient evidence?. *American Journal of Surgery*.

[52] Jukema G. N., Bøhm H. J., Hierholzer G. (1997). Vacuum occlusion: a new concept in treatment of soft tissue and bone infections. *Langenbecks Archiv für Chirurgie. Supplement. Kongressband*.

[53] Obremskey W., Metsemakers W., Schlatterer D. (2020). Musculoskeletal infection in orthopaedic trauma: assessment of the 2018 international consensus meeting on musculoskeletal infection. *The Journal of Bone & Joint Surgery*.

[54] Ramsden A. (2020). *Soft tissue management in Fracture-related Infections*.

[55] On behalf of the Fracture-Related Infection (FRI) group, Metsemakers W. J., Morgenstern M. (2020). General treatment principles for fracture-related infection: recommendations from an international expert group. *Archives of Orthopaedic and Trauma Surgery*.

[56] Govaert G. A. M., Termaat M. F. (2019). Diagnostiek en behandeling van fractuur-gerelateerde infecties. *Nederlands Tijdschrift voor Geneeskunde*.

[57] Chan J. K. K., Ferguson J. Y., Scarborough M., McNally M. A., Ramsden A. J. (2019). Management of post-traumatic osteomyelitis in the lower limb: current state of the art. *Indian Journal of Plastic Surgery*.

[58] Suzuki T., Minehara A., Matsuura T., Kawamura T., Soma K. (2014). Negative-pressure wound therapy over surgically closed wounds in open fractures. *Journal of Orthopaedic Surgery*.

[59] Harris L. G., Richards R. G. (2006). Staphylococci and implant surfaces: a review. *Injury*.

[60] Depypere M., Kuehl R., Metsemakers W. J. (2020). Recommendations for systemic antimicrobial therapy in fracture-related infection: a consensus from an international expert group. *Journal of Orthopaedic Trauma*.

[61] Hellebrekers P., Rentenaar R. J., McNally M. A. (2019). Getting it right first time: the importance of a structured tissue sampling protocol for diagnosing fracture-related infections. *Injury*.

[62] Kale M., Padalkar P., Mehta V. (2017). Vacuum-assisted closure in patients with post-operative infections after instrumented spine surgery: a series of 12 cases. *Journal of Orthopaedic Case Reports*.

[63] Wang J., Zhang H., Wang S. (2015). Application of vacuum sealing drainage in the treatment of internal fixation instrument exposure after early postoperative infection. *Minerva Chirurgica*.

[64] Hotchen A. J., Dudareva M., Ferguson J. Y., Sendi P., McNally M. A. (2019). The bach classification of long bone osteomyelitis. *Bone & Joint Research*.

