# Supplementary material for: The Role of Negative-Pressure Wound Therapy in Patients with Fracture-Related Infection: A Systematic Review and Critical Appraisal
Source: Biomed Res Int. 2021 Oct 19;2021:7742227. doi: 10.1155/2021/7742227 (PMC8548908; doi:10.1155/2021/7742227)
Supplement: Supplementary 1 — Appendix I: syntax. [file 7742227.f1.docx]

**Appendix I: Syntax**

All searches were performed on March 18^th^ 2021

**PubMed** (395 results)

("trauma*"[Title/Abstract] OR "fracture*"[Title/Abstract] OR "fractures, bone"[MeSH Terms]) AND ("negative-pressure wound therapy"[MeSH Terms] OR "negative pressure wound therapy"[Title/Abstract] OR "vacuum assisted closure"[MeSH Terms] OR "vacuum assisted closure" [Title/Abstract] OR "vacuum therapy"[Title/Abstract] OR "NPWT"[Title/Abstract] OR "VAC"[Title/Abstract] OR "vacuum-assisted"[Title/Abstract]) AND ("fracture related infection*"[Title/Abstract] OR "osteomyelitis"[Title/Abstract] OR "infection*"[Title/Abstract] OR "surgical site infection"[Title/Abstract] OR "FRI"[Title/Abstract] OR "SSI"[Title/Abstract] OR "posttraumatic osteomyelitis"[Title/Abstract] OR "osteitis"[Title/Abstract] OR "deep infection*"[Title/Abstract] OR "implant associated infection"[Title/Abstract] OR "infect* nonunion"[Title/Abstract] OR "osteomyelitis"[MeSH Terms] OR "osteitis"[MeSH Terms])

**Embase** (359 results)

(‘trauma*’:ti,ab,kw OR ‘fracture*’:ti,ab,kw OR ‘fracture’/exp) AND (‘vacuum assisted closure’/exp OR ‘negative pressure wound therapy’:ti,ab,kw OR ‘vacuum assisted closure’:ti,ab,kw OR ‘vacuum therapy’:ti,ab,kw OR ‘NPWT’:ti,ab,kw OR ‘VAC’:ti,ab,kw OR ‘vacuum-assisted’:ti,ab,kw) AND (‘fracture related infection*’:ti,ab,kw OR ‘osteomyelitis’:ti,ab,kw OR ‘infection*’:ti,ab,kw OR ‘surgical site infection’:ti,ab,kw OR ‘FRI’:ti,ab,kw OR ‘SSI’:ti,ab,kw OR ‘posttraumatic osteomyelitis’:ti,ab,kw OR ‘osteitis’:ti,ab,kw OR ‘deep infection*’:ti,ab,kw OR 'implant associated infection*':ti,ab,kw OR 'infect* nonunion':ti,ab,kw OR ‘osteomyelitis’/exp OR ‘osteitis’/exp)

Filters: *Sources: ‘Embase’ and ‘Embase and MEDLINE’* and *Publication type: ‘Article’*

**Web of Science** (373 results)

TS=(trauma* OR fracture* OR fracture) AND TS=(vacuum assisted closure OR negative pressure wound therapy OR vacuum therapy OR NPWT OR VAC OR vacuum-assisted) AND TS=(fracture related infection OR osteomyelitis OR infection OR surgical site infection OR FRI OR SSI OR posttraumatic osteomyelitis OR osteitis OR deep infection OR implant associated infection OR infect* nonunion)

Filter: *Publication type: ‘Article’*
