# Supplementary material for: The Role of Negative-Pressure Wound Therapy in Patients with Fracture-Related Infection: A Systematic Review and Critical Appraisal
Source: Biomed Res Int. 2021 Oct 19;2021:7742227. doi: 10.1155/2021/7742227 (PMC8548908; doi:10.1155/2021/7742227)
Supplement: Supplementary 2 — Appendix II: quality assessment. [file 7742227.f2.docx]

**Appendix III: Quality assessment**

The methodological quality of the included studies was assessed using the Newcastle-Ottawa Quality Assessment Scale (NOS), a tool used for assessing the quality of non-randomized cohort and case control studies^29^. The scored criteria are presented below. Case reports were not assessable and therefore marked as ‘N/A’ and excluded from the further analysis. Furthermore, ‘N/A’ was scored due to the absence of a control group and a missing or undescribed follow-up period. Criteria scored ‘N/A’ were not taken into account in the total score. An overall NOS score cutoff of ≥7 stars was chosen for inclusion. A rating overview is presented in Table 1.

**Selection**

1) Representativeness of the exposed cohort

a) truly representative of the average trauma patient with FRI in the community **🟑**

b) somewhat representative of the average trauma patient with FRI in the community **🟑**

c) selected group of users eg nurses, volunteers

d) no description of the derivation of the cohort

2) Selection of the non exposed cohort

a) drawn from the same community as the exposed cohort **🟑**

b) drawn from a different source

c) no description of the derivation of the non exposed cohort

3) Ascertainment of exposure

a) secure record (eg surgical records) **🟑**

b) structured interview **🟑**

c) written self report

d) no description

4) Demonstration that outcome of interest was not present at start of study

a) yes **🟑**

b) no

**Comparability**

1) Comparability of cohorts on the basis of the design or analysis

a) study controls for type of bacteria (mixture/resistance) **🟑**

b) study controls for any additional factor **🟑**

**Outcome**

1) Assessment of outcome

a) independent blind assessment **🟑**

b) record linkage **🟑**

c) self report

d) no description

2) Was follow-up long enough for outcomes to occur

a) yes (≥1 year) **🟑**

b) no

3) Adequacy of follow up of cohorts

a) complete follow up - all subjects accounted for **🟑**

b) subjects lost to follow up unlikely to introduce bias - small number lost 🡪 5% follow up, or

description provided of those lost **🟑**

c) follow up rate < 5% and no description of those lost

d) no statement
